# What has changed and remained the same in the past 55 years regarding the prediction and prevention of postendoscopic retrograde cholangiopancreatography pancreatitis?

**DOI:** 10.1111/den.15025

**Published:** 2025-03-23

**Authors:** Mamoru Takenaka, Masatoshi Kudo

**Affiliations:** ^1^ Department of Gastroenterology and Hepatology Kindai University Faculty of Medicine Osaka‐Sayama Japan

Although 55 years have passed since endoscopic retrograde cholangiopancreatography (ERCP) was first reported in 1968,^1^ post‐ERCP pancreatitis (PEP) remains a major clinical challenge. A systematic review of 108 randomized controlled trials conducted between 1977 and 2012 revealed a 9.7% incidence of PEP among 13,296 patients in the control group.^2^ Further, a prospective multicenter observational study involving 3739 cases reported a PEP incidence of 6.9%.^3^ Notably, several studies have documented an increasing trend in PEP incidence over time. An analysis of 1.22 million hospitalizations in the United States from 2011 to 2017 showed rising hospitalization and fatality rates associated with PEP.^4^ This trend may be attributed to the development and increased use of new therapeutic devices such as metal stents and cholangioscopes over the past 55 years. Meanwhile, there has been significant progress in understanding and preventing PEP, with studies identifying predictive factors and preventive strategies for PEP. Unlike 55 years ago, clinicians can now assess patient‐related and procedure‐related risk factors for PEP before ERCP and consider appropriate preventive measures. However, effective prediction and prevention require a comprehensive understanding of the latest advancements.

In this issue of *Digestive Endoscopy*, Kato *et al*. present an in‐depth review titled “Current status and issues for prediction and prevention of post‐endoscopic retrograde cholangiopancreatography pancreatitis.”^5^ This article explores the latest prediction models, scoring systems, and newly identified patient factors. The following is a summary of changes and enduring aspects regarding PEP over the past 55 years.

## RISK FACTORS FOR PEP

In the early days of ERCP, knowledge about the risk factors for PEP was limited. There was no awareness that groups, such as young women, were at higher risk of developing PEP. Today, extensive research has identified various risk factors, often broadly categorized as patient‐related factors (e.g. younger age or female sex) and procedure‐related factors (e.g. difficult cannulation, repeated pancreatic duct instrumentation, or pancreatic duct guidewire placement). In recent years, anatomical factors such as large pancreatic parenchymal volume, high pancreatic fat content, and specific duodenal papilla morphology have been reported as risk factors for PEP.

As also emphasized by Kato *et al*.,^5^ it is rare for patients undergoing ERCP to possess only a single risk factor, and several studies have reported the prediction of PEP using a prediction model and scoring system. In addition, artificial intelligence has been reported to be a helpful option for dealing with the increasingly complex risk factors of PEP in an integrated manner. The development of PEP prediction models has been reported, with further growth expected.

## PREVENTIVE STRATEGIES FOR PEP

Over the decades, three interventions, such as nonsteroidal antiinflammatory drugs (NSAIDs), pancreatic stent placement (PSP), and aggressive hydration, have emerged as standard preventive measures for PEP.

Rectally administered NSAIDs, such as diclofenac and indomethacin, have been shown to be highly effective in reducing the risk of PEP, with several randomized controlled trials and meta‐analyses supporting their use. Guidelines in the United States, Europe, and Japan are now recommend rectally administered NSAIDs. However, there is ongoing debate regarding the optimal dosage, as the standard 100 mg dose recommended in guidelines differs from the lower doses commonly used in some countries, including Japan. PSP is employed in patients with a particularly high risk of PEP.^6^ Comparative studies have demonstrated that combining NSAIDs with PSP further reduces the risk of PEP compared to NSAIDs alone. Toyonaga *et al*. also reported that PSP may reduce the risk of PEP following the placement of covered self‐expandable metal stents.^7^

More recently, aggressive fluid replacement has been identified as a potential PEP prevention strategy. While promising, variability exists in the protocols regarding the volume, timing, and rate of fluid administration, and further research is needed to establish standardized guidelines.

Kato *et al*.^5^ also discussed novel PEP prevention measures such as the use of tacrolimus or nitrate as a drug in combination with NSAIDs, the precut technique, and facility and operator factors. Tacrolimus is a calcineurin inhibitor, and when used in combination with NSAIDs, it is effective in preventing PEP. Animal experiments have also shown that it is effective when administered rectally or via the pancreas. However, currently, the immunosuppressant tacrolimus is not used in healthy people.

Nitrates, especially when administered sublingually, are also reported to have a preventive effect on PEP; however, there is no sufficient supporting evidence.

Despite several reports of the usefulness of precut, the timing of trans‐pancreatic sphincterotomy and its preventive effect on PEP need to be verified.

## IMPACT OF NEW DEVICES AND TREATMENT TECHNIQUES ON PEP

Over the past 55 years, technological advancements have resulted in new therapeutic devices, such as metal stents and cholangioscopes, which have enhanced ERCP's diagnostic and therapeutic capabilities. Recent studies suggest a potential increase in PEP cases owing to the broader use of complex therapeutic procedures and devices.

Additionally, endoscopic ultrasound‐guided biliary drainage (EUS‐BD) has emerged as an alternative to ERCP.^8^, ^9^ Unlike ERCP, EUS‐BD does not carry the risk of postoperative pancreatitis; however, its adoption remains limited due to technical complexity and the need for specialized facilities and expertise. Despite this, compared to the early days of ERCP, there is currently a greater need to carefully consider the indications for ERCP, while also keeping in mind the indications for EUS‐BD. Furthermore, to enable better indication selection, it is necessary to clarify the impact of new devices and techniques on PEP.

## BIOMARKERS FOR EARLY DETECTION

There have also been advancements in the early diagnosis of PEP. Measuring pancreatic enzyme activity (e.g. serum amylase or lipase) within a few hours of ERCP is standard practice. Hirota *et al*. analyzed 20 articles to evaluate the usefulness of pancreatic enzyme levels using a pooled analysis and concluded that serum amylase or lipase levels more than three times the upper limit of normal measured 2–4 h after ERCP are helpful for predicting PEP.^10^

In addition, novel biomarkers such as urinary trypsinogen‐2 have shown promise for rapid and accurate early detection. These developments allow for quicker intervention, potentially reducing the severity of PEP.

Prevention of PEP is a top priority for clinicians performing ERCP. While the methods have evolved, the need to minimize risk through procedural care, patient assessment, and preventive interventions remains constant.

Over the past five decades, significant advancements have been made in the understanding, prediction, and prevention of PEP. The identification of risk factors, development of preventive strategies such as NSAIDs and PSP, and introduction of predictive models and biomarkers represent important progress. However, challenges persist, including the rising incidence of PEP in some contexts, variability in preventive protocols, and the need for further research into alternative techniques such as EUS‐BD. Although ERCP remains the cornerstone of managing biliary‐pancreatic disease, the increasing adoption of EUS‐BD is controversial. Technological advances have increased the complexity of ERCP and place greater responsibility on the clinician than they did 55 years ago.

Careful patient selection remains as critical as it was 55 years ago. ERCP should only be performed when clearly indicated, as unnecessary procedures expose patients to avoidable risks, including PEP. This principle continues to be a cornerstone of responsible clinical practice. The responsibility of medical professionals to perform ERCP with care and diligence also remains unchanged. Clinicians must stay updated on best practices, adopt new evidence‐based techniques, and always prioritize patient safety. Awareness of PEP risk factors and preventive strategies is as critical today as it was 55 years ago.

As ERCP continues to be an essential tool in gastroenterology, clinicians must remain vigilant and informed and strive to reduce the risk of complications and improve patient outcomes. While the field has made great strides, the ongoing commitment to safety and innovation will define the next 55 years of progress in ERCP practice.

## CONFLICT OF INTEREST

Author M.T. is an Associate Editor of *Digestive Endoscopy*. The other author declares no conflict of interest for this article.

## FUNDING INFORMATION

None.
